# 2-(2-Bromo­phen­yl)acetic acid

**DOI:** 10.1107/S1600536812020545

**Published:** 2012-05-12

**Authors:** Rajni Kant, Kamini Kapoor, B. Narayana

**Affiliations:** a X-ray Crystallography Laboratory, Post-Graduate Department of Physics & Electronics, University of Jammu, Jammu Tawi 180 006, India; b Department of Studies in Chemistry, Mangalore University, Mangalagangotri 574 199, India

## Abstract

In the title mol­ecule, C_8_H_7_BrO_2_, the carboxyl group is twisted by 76.2 (3)° from the benzene ring plane. In the crystal, mol­ecules are linked into inversion dimers through pairs of O—H⋯O hydrogen bonds. The dimers are further linked into layers parallel to the *bc* plane by weak C—H⋯O hydrogen bonds.

## Related literature

For applications of the title compound, see: Deshpande *et al.* (2008 ▶); Rodriguesa *et al.* (2002 ▶); Pratt *et al.* (2000 ▶). For related structures, see: Hodgson & Asplund (1991 ▶); Harris *et al.* (1994 ▶); Hartung *et al.* (2004 ▶); Yuan *et al.* (2008 ▶); Jasinski *et al.*(2010 ▶); Li *et al.* (2010 ▶).
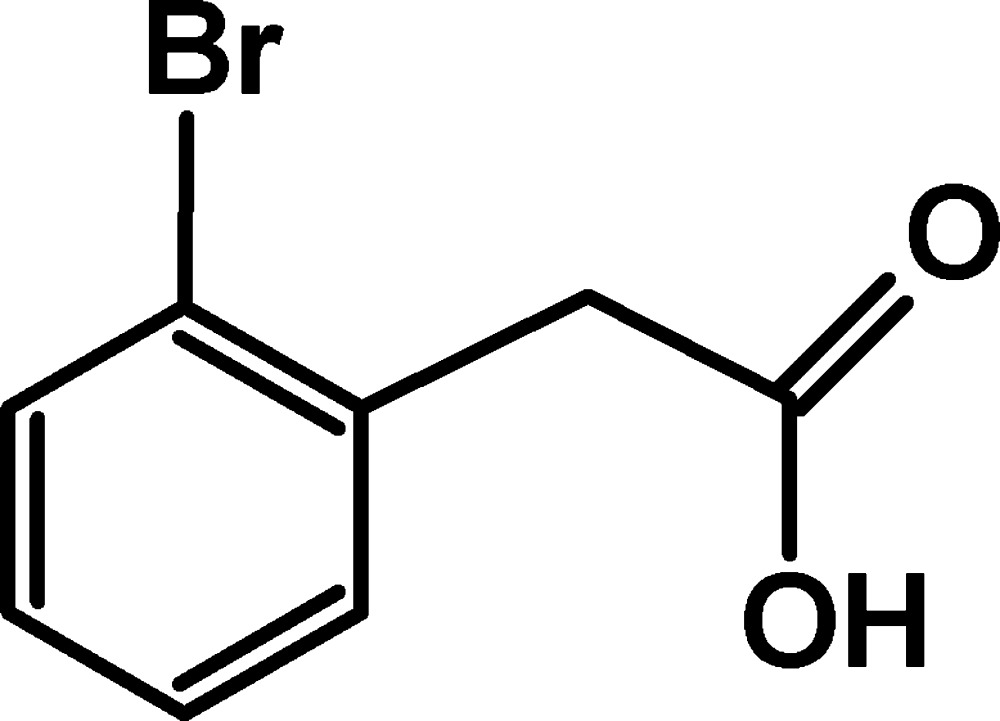


## Experimental

### 

#### Crystal data

C_8_H_7_BrO_2_
*M* *_r_* = 215.05
Monoclinic, 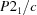

*a* = 8.9732 (5) Å
*b* = 5.9114 (3) Å
*c* = 15.8489 (7) Å
β = 99.529 (5)°
*V* = 829.09 (7) Å^3^
*Z* = 4
Mo *K*α radiation
μ = 4.90 mm^−1^
*T* = 293 K
0.3 × 0.2 × 0.2 mm

#### Data collection

Oxford Diffraction Xcalibur Sapphire3 diffractometer
Absorption correction: multi-scan (*CrysAlis PRO*; Oxford Diffraction, 2010 ▶) *T*_min_ = 0.370, *T*_max_ = 1.000
8471 measured reflections
1628 independent reflections
1248 reflections with *I* > 2σ(*I*)
*R*_int_ = 0.043

#### Refinement

*R*[*F* ^2^ > 2σ(*F* ^2^)] = 0.042
*wR*(*F* ^2^) = 0.096
*S* = 1.07
1628 reflections
105 parameters
H atoms treated by a mixture of independent and constrained refinement
Δρ_max_ = 0.55 e Å^−3^
Δρ_min_ = −0.69 e Å^−3^

### 

Data collection: *CrysAlis PRO* (Oxford Diffraction, 2010 ▶); cell refinement: *CrysAlis PRO*; data reduction: *CrysAlis PRO*; program(s) used to solve structure: *SHELXS97* (Sheldrick, 2008 ▶); program(s) used to refine structure: *SHELXL97* (Sheldrick, 2008 ▶); molecular graphics: *ORTEP-3* (Farrugia, 1997) ▶; software used to prepare material for publication: *PLATON* (Spek, 2009 ▶).

## Supplementary Material

Crystal structure: contains datablock(s) I, global. DOI: 10.1107/S1600536812020545/cv5295sup1.cif

Structure factors: contains datablock(s) I. DOI: 10.1107/S1600536812020545/cv5295Isup2.hkl

Supplementary material file. DOI: 10.1107/S1600536812020545/cv5295Isup3.cml

Additional supplementary materials:  crystallographic information; 3D view; checkCIF report

## Figures and Tables

**Table 1:** Hydrogen-bond geometry (Å, °)

*D*—H⋯*A*	*D*—H	H⋯*A*	*D*⋯*A*	*D*—H⋯*A*
O8—H8⋯O9^i^	0.87 (7)	1.76 (7)	2.630 (4)	175 (3)
C6—H6⋯O9^ii^	0.93	2.57	3.453 (5)	158

Symmetry codes: (i) 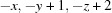; (ii) 
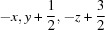
.

**Fig. 1.**
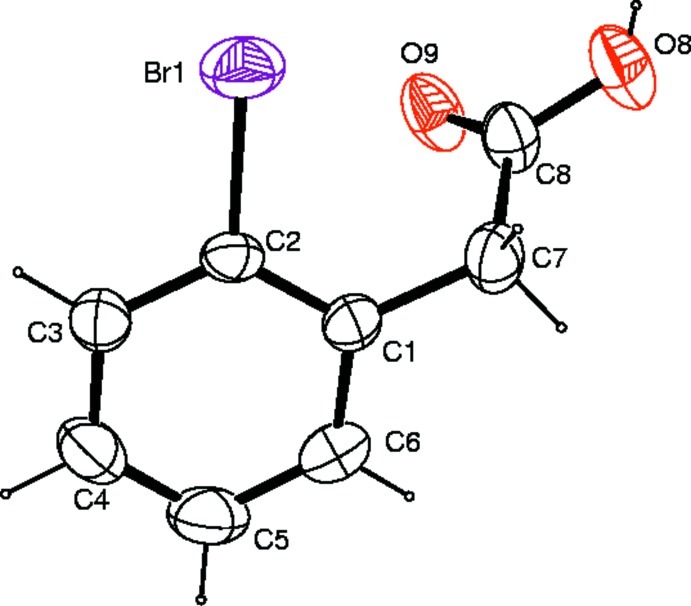
ORTEP view of the molecule with the atom-labeling scheme. The Displacement ellipsoids are drawn at the 40% probability level. H atoms are shown as small spheres of arbitrary radii.

## supplementary crystallographic information

### Comment

The title compound, (I), has many applications in various syntheses, for example, in the synthesis of heterocyclic compounds (Deshpande *et al.*, 2008) and in the synthesis of AMPA antagonists - substituted quinoxalinediones, quinolones, isatinoximes and benzodiazepine derivatives (Pratt *et al.*, 2000). It can also be used in a gas chromatography as a phase transfer catalyst (Rodriguesa *et al.*, 2002). Herein we report its crystal structure.

In (I) (Fig. 1), all bond lengths and angles are normal and correspond to those observed in related structures (Hodgson & Asplund,1991; Harris *et al.*, 1994; Hartung *et al.*, 2004; Yuan *et al.*, 2008; Jasinski *et al.*, 2010; Li *et al.*, 2010). The carboxyl group is twisted at 76.2 (3)° from the benzene ring plane. Intermolecular O—H···O hydrogen bonds (Table 1) link the molecules into centrosymmetric dimers. Weak intermolecular C—H···O interactions (Table 1) link further these dimers into layers parallel to *bc* plane.

### Experimental

The title compound was purchased from the Spectrochem Ltd., Mumbai. Single-crystals were grown from acetone and toluene(1:1) mixture by slow evaporation method.

### Refinement

Atom H8 was found in a difference map and isotropically refined. C-bound H atoms were positioned geometrically (C—H 0.93–0.97 Å), and were treated as riding, with *U*_iso_(H) = 1.2*U*_eq_(C).

### Crystal data

**Table:**

C_8_H_7_BrO_2_	*F*(000) = 424
*M**_r_* = 215.05	*D*_x_ = 1.723 Mg m^−^^3^
Monoclinic, *P*2_1_/*c*	Melting point = 381–377 K
Hall symbol: -P 2ybc	Mo *K*α radiation, λ = 0.71073 Å
*a* = 8.9732 (5) Å	Cell parameters from 3025 reflections
*b* = 5.9114 (3) Å	θ = 3.4–29.1°
*c* = 15.8489 (7) Å	µ = 4.90 mm^−^^1^
β = 99.529 (5)°	*T* = 293 K
*V* = 829.09 (7) Å^3^	Prism, white
*Z* = 4	0.3 × 0.2 × 0.2 mm

### Data collection

**Table:**

Oxford Diffraction Xcalibur Sapphire3 diffractometer	1628 independent reflections
Radiation source: fine-focus sealed tube	1248 reflections with *I* > 2σ(*I*)
Graphite monochromator	*R*_int_ = 0.043
Detector resolution: 16.1049 pixels mm^-1^	θ_max_ = 26.0°, θ_min_ = 3.7°
ω scan	*h* = −11→11
Absorption correction: multi-scan (*CrysAlis PRO* RED; Oxford Diffraction, 2010)	*k* = −7→7
*T*_min_ = 0.370, *T*_max_ = 1.000	*l* = −19→19
8471 measured reflections	

### Refinement

**Table:**

Refinement on *F*^2^	Secondary atom site location: difference Fourier map
Least-squares matrix: full	Hydrogen site location: inferred from neighbouring sites
*R*[*F*^2^ > 2σ(*F*^2^)] = 0.042	H atoms treated by a mixture of independent and constrained refinement
*wR*(*F*^2^) = 0.096	*w* = 1/[σ^2^(*F*_o_^2^) + (0.0324*P*)^2^ + 1.055*P*] where *P* = (*F*_o_^2^ + 2*F*_c_^2^)/3
*S* = 1.07	(Δ/σ)_max_ = 0.001
1628 reflections	Δρ_max_ = 0.55 e Å^−^^3^
105 parameters	Δρ_min_ = −0.69 e Å^−^^3^
0 restraints	Extinction correction: *SHELXL97* (Sheldrick, 2008), Fc^*^=kFc[1+0.001xFc^2^λ^3^/sin(2θ)]^-1/4^
Primary atom site location: structure-invariant direct methods	Extinction coefficient: 0.071 (3)

### Special details

**Table:**

Experimental. *CrysAlis PRO*, Oxford Diffraction Ltd., Version 1.171.34.40 (release 27–08-2010 CrysAlis171. NET) (compiled Aug 27 2010,11:50:40) Empirical absorption correction using spherical harmonics, implemented in SCALE3 ABSPACK scaling algorithm.
Geometry. All e.s.d.'s (except the e.s.d. in the dihedral angle between two l.s. planes) are estimated using the full covariance matrix. The cell e.s.d.'s are taken into account individually in the estimation of e.s.d.'s in distances, angles and torsion angles; correlations between e.s.d.'s in cell parameters are only used when they are defined by crystal symmetry. An approximate (isotropic) treatment of cell e.s.d.'s is used for estimating e.s.d.'s involving l.s. planes.
Refinement. Refinement of *F*^2^ against ALL reflections. The weighted *R*-factor *wR* and goodness of fit *S* are based on *F*^2^, conventional *R*-factors *R* are based on *F*, with *F* set to zero for negative *F*^2^. The threshold expression of *F*^2^ > σ(*F*^2^) is used only for calculating *R*-factors(gt) *etc*. and is not relevant to the choice of reflections for refinement. *R*-factors based on *F*^2^ are statistically about twice as large as those based on *F*, and *R*- factors based on ALL data will be even larger.

### Fractional atomic coordinates and isotropic or equivalent isotropic displacement parameters (Å^2^)

**Table:**

	*x*	*y*	*z*	*U*_iso_*/*U*_eq_	
Br1	0.42140 (6)	0.23223 (8)	0.92648 (3)	0.0681 (3)	
C1	0.2498 (4)	0.4863 (6)	0.7908 (2)	0.0427 (9)	
C2	0.3352 (4)	0.2936 (6)	0.8109 (2)	0.0395 (8)	
C3	0.3640 (4)	0.1404 (7)	0.7497 (3)	0.0498 (10)	
H3	0.4208	0.0110	0.7654	0.060*	
C4	0.3078 (6)	0.1819 (8)	0.6660 (3)	0.0627 (12)	
H4	0.3275	0.0813	0.6241	0.075*	
C5	0.2223 (6)	0.3715 (9)	0.6430 (3)	0.0689 (13)	
H5	0.1839	0.3990	0.5857	0.083*	
C6	0.1935 (5)	0.5208 (7)	0.7051 (3)	0.0599 (12)	
H6	0.1347	0.6480	0.6890	0.072*	
C7	0.2239 (5)	0.6574 (7)	0.8568 (3)	0.0570 (11)	
H7A	0.3211	0.7023	0.8887	0.068*	
H7B	0.1779	0.7906	0.8276	0.068*	
O8	0.1337 (4)	0.7126 (6)	0.9850 (2)	0.0710 (10)	
C8	0.1266 (4)	0.5795 (7)	0.9188 (3)	0.0520 (10)	
O9	0.0457 (3)	0.4130 (5)	0.9081 (2)	0.0650 (9)	
H8	0.072 (7)	0.678 (10)	1.020 (5)	0.11 (2)*	

### Atomic displacement parameters (Å^2^)

**Table:**

	*U*^11^	*U*^22^	*U*^33^	*U*^12^	*U*^13^	*U*^23^
Br1	0.0835 (4)	0.0712 (4)	0.0440 (3)	−0.0020 (2)	−0.0059 (2)	0.0091 (2)
C1	0.049 (2)	0.0354 (19)	0.047 (2)	−0.0069 (16)	0.0158 (18)	0.0034 (16)
C2	0.045 (2)	0.041 (2)	0.0330 (19)	−0.0082 (16)	0.0085 (16)	0.0050 (15)
C3	0.058 (2)	0.043 (2)	0.052 (3)	−0.0005 (18)	0.021 (2)	0.0007 (19)
C4	0.085 (3)	0.059 (3)	0.049 (3)	−0.015 (2)	0.026 (2)	−0.014 (2)
C5	0.088 (3)	0.079 (3)	0.038 (3)	−0.007 (3)	0.004 (2)	0.007 (2)
C6	0.070 (3)	0.053 (2)	0.055 (3)	0.007 (2)	0.006 (2)	0.016 (2)
C7	0.070 (3)	0.040 (2)	0.067 (3)	−0.005 (2)	0.029 (2)	−0.004 (2)
O8	0.080 (2)	0.069 (2)	0.071 (2)	−0.0264 (17)	0.0341 (19)	−0.0325 (17)
C8	0.053 (2)	0.048 (2)	0.058 (3)	−0.0015 (19)	0.019 (2)	−0.010 (2)
O9	0.0697 (19)	0.0631 (19)	0.069 (2)	−0.0253 (16)	0.0323 (16)	−0.0258 (16)

### Geometric parameters (Å, º)

**Table:**

Br1—C2	1.901 (4)	C5—C6	1.378 (6)
C1—C2	1.380 (5)	C5—H5	0.9300
C1—C6	1.383 (5)	C6—H6	0.9300
C1—C7	1.502 (5)	C7—C8	1.491 (6)
C2—C3	1.383 (5)	C7—H7A	0.9700
C3—C4	1.361 (6)	C7—H7B	0.9700
C3—H3	0.9300	O8—C8	1.304 (5)
C4—C5	1.373 (7)	O8—H8	0.87 (7)
C4—H4	0.9300	C8—O9	1.218 (5)
			
C2—C1—C6	116.6 (4)	C6—C5—H5	120.2
C2—C1—C7	122.6 (4)	C5—C6—C1	121.7 (4)
C6—C1—C7	120.8 (4)	C5—C6—H6	119.1
C1—C2—C3	122.6 (4)	C1—C6—H6	119.1
C1—C2—Br1	120.1 (3)	C8—C7—C1	115.2 (3)
C3—C2—Br1	117.2 (3)	C8—C7—H7A	108.5
C4—C3—C2	118.9 (4)	C1—C7—H7A	108.5
C4—C3—H3	120.5	C8—C7—H7B	108.5
C2—C3—H3	120.5	C1—C7—H7B	108.5
C3—C4—C5	120.4 (4)	H7A—C7—H7B	107.5
C3—C4—H4	119.8	C8—O8—H8	115 (4)
C5—C4—H4	119.8	O9—C8—O8	123.3 (4)
C4—C5—C6	119.7 (4)	O9—C8—C7	123.9 (4)
C4—C5—H5	120.2	O8—C8—C7	112.8 (4)
			
C6—C1—C2—C3	−0.2 (5)	C4—C5—C6—C1	0.6 (7)
C7—C1—C2—C3	−177.4 (3)	C2—C1—C6—C5	−0.6 (6)
C6—C1—C2—Br1	178.8 (3)	C7—C1—C6—C5	176.8 (4)
C7—C1—C2—Br1	1.5 (5)	C2—C1—C7—C8	−68.9 (5)
C1—C2—C3—C4	0.9 (6)	C6—C1—C7—C8	114.0 (4)
Br1—C2—C3—C4	−178.1 (3)	C1—C7—C8—O9	−15.8 (6)
C2—C3—C4—C5	−0.9 (6)	C1—C7—C8—O8	166.1 (4)
C3—C4—C5—C6	0.2 (7)		

### Hydrogen-bond geometry (Å, º)

**Table:**

*D*—H···*A*	*D*—H	H···*A*	*D*···*A*	*D*—H···*A*
O8—H8···O9^i^	0.87 (7)	1.76 (7)	2.630 (4)	175 (3)
C6—H6···O9^ii^	0.93	2.57	3.453 (5)	158

Symmetry codes: (i) −*x*, −*y*+1, −*z*+2; (ii) −*x*, *y*+1/2, −*z*+3/2.
